# Partnering for change (P4C) in Sweden- a study protocol of a collaborative school-based service delivery model to create inclusive learning environments

**DOI:** 10.1186/s12889-023-17053-0

**Published:** 2023-11-10

**Authors:** Vedrana B. Baric, Moa Yngve, Marie Holmefur, Inna Feldman, Jenny Wilder, Kine Johansen, Nina Klang, Helene Lidström, Maria Borgestig

**Affiliations:** 1 Department of Health, Medicine and Caring Sciences, Linköping University, Linköping, Sweden; 2 Department of Women’s and Children’s Health, Uppsala University, Uppsala, Sweden; 3 School of Health Sciences, Örebro University, Örebro, Sweden; 4 Department of Public Health and Caring Sciences, Uppsala University, Uppsala, Sweden; 5 Department of Special Education, Stockholm University, Stockholm, Sweden; 6 Department of Education, Uppsala University, Uppsala, Sweden; 7 School of Education, Culture and Communication, Mälardalen University, Vasteras, Sweden

**Keywords:** Children with special needs, Dynamic performance analysis (DPA), Inclusive learning environments, Mental health, Partnering for change (P4C), Response to intervention (RtI), School-based service delivery model

## Abstract

**Background:**

Inclusive learning environments are considered as crucial for children’s engagement with learning and participation in school. Partnering for change (P4C) is a collaborative school-based service delivery model where services are provided at three levels of intensity based on children’s needs (class, group-, individual interventions). Interventions in P4C are provided universally to support all children with learning, not only children with special education needs (SEN), and as such are expected to be health-promoting.

**Aim:**

The aim of the study is to evaluate the effectiveness and cost-effectiveness of P4C as well as school staff members’ and children’s experiences after P4C.

**Methods:**

In a parallel, non-randomised controlled intervention design, 400 children, aged 6–12 years, and their teachers, will be recruited to either intervention classes, working according to the P4C, or to control classes (allocation ratio 1:1). Data will be collected at baseline, post-intervention (4 months), and 11 months follow-up post baseline. The primary outcome is children’s engagement with learning in school. Secondary outcomes include for example children’s health-related quality of life and wellbeing, occupational performance in school, attendance, and special educational needs. The difference-in-differences method using regression modelling will be applied to evaluate any potential changes following P4C. Focus group interviews focusing on children, and professionals’ experiences will be performed after P4C. A health economic evaluation of P4C will be performed, both in the short term (post intervention) and the long term (11-month follow-up). This study will provide knowledge about the effectiveness of P4C on children’s engagement with learning, mental health, and wellbeing, when creating inclusive learning environments using a combination of class-, group- and individual-level interventions.

**Trial registration number:**

NCT05435937.

**Supplementary Information:**

The online version contains supplementary material available at 10.1186/s12889-023-17053-0.

## Introduction

Participation in school is considered important for both learning and development, as well as for health and well-being to ensure that children reach their full potential across their lifespan [1]. It is widely recognised that engagement is related to participation and children’s learning across academic, social–emotional, and behavioural domains [2–5]. Children with poor engagement in school are more likely to experience difficulties with schoolwork, have fewer positive relationships with teachers, higher rates of bullying behaviour, risky health behaviours, and mental health problems [2]. Reports show that the proportion of school-aged children experiencing mental health problems is increasing [6], and is more common among children with disabilities [7, 8]. There is a clear link between mental health problems and restricted participation in school [9, 10]. For example, children with disabilities such as neurodevelopmental disorders (e.g., attention-deficit hyperactivity disorder (ADHD), autism spectrum disorder, conduct disorder) often report lower engagement with learning than their peers, and difficulties meeting educational requirements [11]. All these problems entail high costs for individuals, families, and for society at large [12]. Non-inclusive learning environments are considered to restrict children’s engagement with learning in school leading to difficulties meeting educational goals [13–17]. Thus, it is pivotal for schools to create inclusive learning environments to enhance engagement and participation. Identifying and providing interventions for children at risk of poor engagement with learning and restricted participation may have a beneficial effect on children’s mental health and well-being [11, 18, 19].

Partnering for change (P4C) is an innovative service delivery model aiming to enhance inclusive learning environments by providing a combination of class-, group- and individual-level interventions. Our hypothesis, supported by international research [20–22], is that implementing P4C will promote engagement with learning and well-being for all children, as well as help prevent mental health problems. P4C aims to create inclusive environments through capacity building, partnerships, and interdisciplinary collaboration in multiprofessional teams at schools [22]. Interventions according to P4C are built on universal design for learning (UDL) and are delivered according to a tiered approach on class-, group- and individual-levels. Hence, interventions focus both on school-wide interventions aiming to improve the learning environment for all children [23], as well as targeted intervention for children demonstrating low engagement with learning and participation restrictions in school [24–26]. Previous studies have shown promising results in P4C to accurately identify children in need of support [21, 27], positively influencing family–therapist relationships [28], increasing knowledge and capacity among teachers, parents, and occupational therapists (OTs) to identify and tailor support based on children’s needs [22, 29]. Additionally, P4C has proven to be effective in providing class-level interventions targeting the entire educational setting, which is important for health promotion [20]. However, there is limited research on P4C’s effectiveness on children’s engagement across social–emotional and behavioural domains. Additionally, the evidence on the cost-effectiveness of P4C is lacking. While P4C has been implemented and evaluated in Canada with promising results [21, 22, 27, 29], the service delivery model has yet to be implemented in Sweden. A previous study on the feasibility of P4C shows promising results concerning its implementation and acceptability (Yngve et al., in manuscript). To promote implementation in Sweden, additional results are needed on the effectiveness of P4C on children’s engagement with learning, well-being, and occupational performance in school and its cost-effectiveness.

### Aim

The aim of the study is to evaluate the effectiveness and cost-effectiveness of P4C as well as school staff members’ and children’s experiences after P4C. The study addresses the following research questions:

- What is the effectiveness of P4C on children’s: (a) engagement with learning, (b) health-related quality of life and well-being, (c) occupational performance, (d) attainment of school-based occupational performance goals, and (e) school attendance, (f) student–environment fit, and (g) number of children with special educational needs?
- Is P4C a cost-effective intervention compared to the control condition?
- How does P4C affect the school staff’s knowledge, skills, and experiences of P4C and children’s diverse needs?
- How do children in intervention classes experience their learning environment?

## Methods: participants, interventions, and outcomes

### Study design

**Fig. 1:**
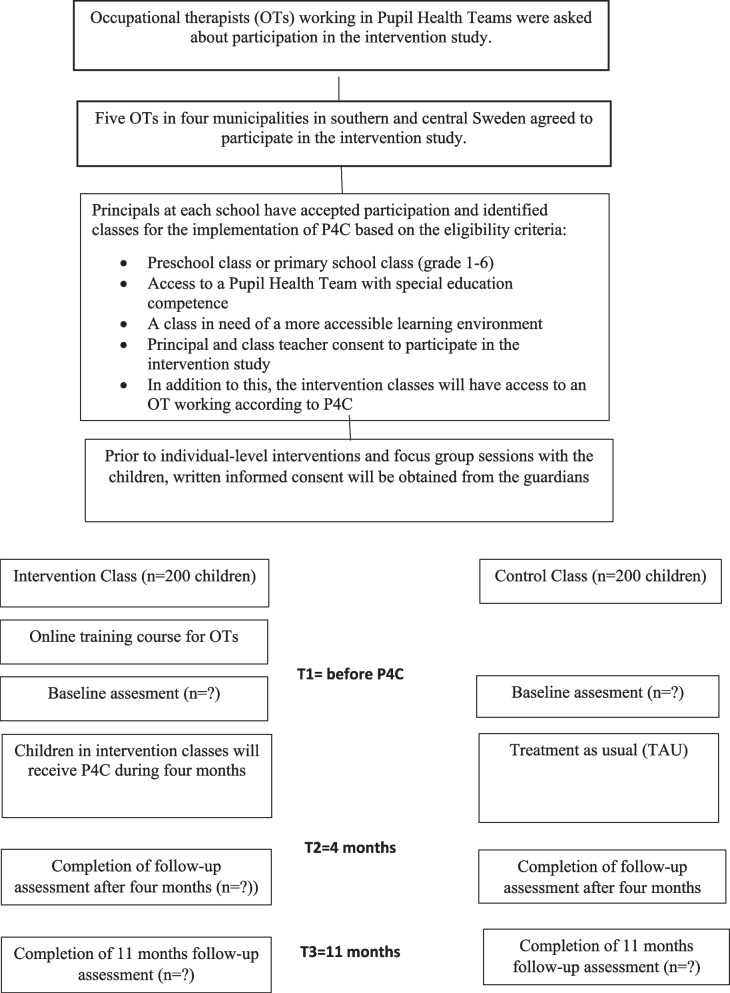
Time schedule of enrolment, interventions and assessments. P4C, Partnering for Change; OT, Occupational therapist, TAU Treatment as usual

The study will be conducted as a parallel, non-randomised controlled intervention study, with pre-, post- and 11-month follow-ups. It will include a qualitative evaluation involving interviews with school staff and children after P4C. The study will be conducted in elementary schools in Sweden during one school semester (approximately four months). In Swedish elementary schools, all pupils have access to Pupil Health Teams (PHTs), which will constitute treatment as usual (TAU). Intervention classes will also work according to P4C. Study design and enrolment details are presented in Fig. 1. This protocol is reported in accordance with the Standard Protocol Items: Recommendations for Interventional Trials (SPIRIT) [30] (Supplemental files 1). The intervention study has been approved by the Swedish Ethical Review Authority (2023-00013-02, 2021-06412-02, 2019-03954), and registered as a clinical trial at ClinicalTrials.gov (NCT05435937).

### Study setting and eligibility criteria

The intervention study will be conducted in elementary schools that include classes from preschool to sixth grade. Inclusion criteria for intervention- and control classes are: (a) preschool class or primary school class (grades 1–6), (b) have a Pupil Health Team with special education competence, (c) there are children with unmet special educational needs as perceived by the school staff, and (d) the principal and class teacher both consent to participate in the intervention study. The intervention classes will have access to an OT working according to P4C. OTs working according to P4C must have attended an online training course. Approximately 400 children in total (intervention and control, allocation ratio 1:1) will participate in the study.

### Recruitment

Information about OTs working in Pupil Health Teams in Swedish elementary schools will be retrieved using publicly available information on municipal websites and a closed Facebook group for OTs. Identified OTs will be provided with information about the intervention study, including study aims, the online training course in P4C, and the data collection methods—either via personal e-mail from the authors or via posts in the Facebook group. Principals, teachers, and/or special education teachers will be provided with information about the study by the OTs. Control classes will be recruited separately by contacting teachers and principals in different geographical areas. The control classes will be matched to the intervention classes based on class grade, gender, and socio-geographical area.

### Intervention classes receiving P4C

P4C is based on close collaboration, partnership, and knowledge translation (capacity building) between teachers and OTs, and is intended to create a sustainable inclusive learning environment that enhance children’s engagement with learning and participation [21, 22]. P4C is delivered based on the three-tiered approach, in which class-level interventions based on UDL, targeting the whole class, are implemented first, after which follow group-level interventions. Finally, when interventions in the first and second tier are not enough to engage all children in schoolwork, individual-level interventions are provided.

 Before applying P4C, participating OTs will attend an online training course consisting of seven digital modules, originally developed by the CanChild research group [22]. The modules were partly adapted to the Swedish school system. Furthermore, ongoing mentorship among participating OTs via web-based meetings will be arranged. For further details about P4C, please see Table 1.

**Table 1:** Intervention-P4C

NAME	Partnering for Change in Sweden (P4C-Swe)
WHY	Capacity building through collaboration and coaching is essential for creating an inclusive learning environment. The core activities that make up this approach include relationship building, knowledge translation, and the three-tiered intervention approach.
WHAT *Materials* *Procedures*	Online training course for OTs *(Missiuna et al, 2012).* ‐ Includes seven modules (M1-M7) online (requires a license, available at: https://canchild.ca/en/partnering-for-change-ot-module ) ‐ Modules content: The P4C model, Universal Design for Learning, Consultative Collaboration; Dynamic Performance Analysis (DPA); Interventions using the three-tiered approach; Promoting sustainable change through knowledge translation, The school system - Each module includes readings, short videos, and learning tasks. - Access is provided to videos created by the research group specifically tailored to the Swedish context. This access also includes learning objectives and activities presented in Swedish, as well as references to websites and texts, such as a description of the Swedish school system. *1. An online training course for Occupational Therapists (OTs):* commences with a startup online discussion meeting. During this meeting, the research group introduces the P4C model and the course tailored for OTs applying the P4C approach. Subsequently, OTs individually study the seven modules (M1-M7) online. Throughout the online course, web-based discussion meetings are conducted and moderated by the research group to enhance the learning experience. Each of these forums lasts for two hours. *2. Delivering P4C (during a semester, approximately 4 months):* P4C is carried out by a multiprofessional team consisting of OT, teachers, and special education teachers in tier 1-3. Tier 1. Class level (Universal design for learning, UDL): The OT observes the class using DPA to identify children at risk of experiencing difficulties with occupational performance in school-based occupations (performance breakdown). Subsequently, the OT and teachers jointly discuss and analyze the causes of non-performance, establish goals and implement adjustments/interventions at the UDL level to enhance occupational performance. The teacher implements the agreed upon interventions, followed by further observations by the OT to evaluate the outcome. If, after implemented UDL interventions, there are still children with difficulties in carrying out current school-based occupations, P4C will proceed to Tier 2. Tier 2. Group level (Differentiated instruction): If, after implementing UDL interventions, there are still children experiencing performance breakdowns, the OT conducts new observations based on DPA. Working in collaboration with teachers, the OT then implements interventions for a group of children, followed by subsequent observations by the OT. Tier 3. Individual level (Accommodations): After Tier 1 and 2, if children continue to experience performance breakdowns, the OT collaborates with the teachers to identify and implement individual-level interventions. *3. Web meetings:* Occur once a month and involve OTs, teachers, and special education teachers collaborating with the research group to monitor and support the implementation of P4C.
WHO PROVIDE	The OT, a part of the schools' Pupil Health Team, collaborates with teachers to implement adjustments and support using the P4C.
HOW AND WHERE	Observations: take place in natural settings in schools. Collaboration: occurs both on school premises and via web meetings.

During the intervention period, the OT will collaborate closely with teachers in intervention classes. The OT will be present in the classroom (about 1/2 day a week). The Dynamic Performance Analysis (DPA) [31] will be used to identify children experiencing difficulties with occupational performance in school (performance break downs). The OT and teacher will jointly analyse the observed difficulties, formulate school-based occupational performance goals, and in collaboration, decide and follow up on interventions needed to prevent or overcome low engagement with learning in school.

### Control classes

According to the Educational Act [32], schools are obliged to identify needs and provide and evaluate extra adaptations (e.g., cognitive support, adapted instructions) in regular classrooms. If the adaptations are not enough, a child may be provided special support including regular contact with a special education teacher, pupil’s assistant, and/or placement in a special teaching group. Children in control classes will receive TAU provided by the Pupil Health Team, which most often consists of adaptions/support provided by a school doctor, school nurse, or psychologist, as well as staff with special educational competence.

### School and public involvement

#### Reference group

A reference group, comprised of representatives from organisations at different levels of government in Sweden, such as the Swedish Association of Local Authorities and Regions, and the National Agency for Special Needs Education and interest organisations will be constituted. The reference group will be involved in discussions concerning methodological issues, interpretation, and disseminating the results. The reference group will meet on a regular basis throughout the study period.

### Outcome measures

Data collection will occur at: (i) baseline, prior to the intervention (T1); (ii) immediately following the intervention (T2); and (iii) eleven months follow-up post-baseline (T3). An overview of all outcome measures and data collection points is presented in Table 2.

**Table 2:**
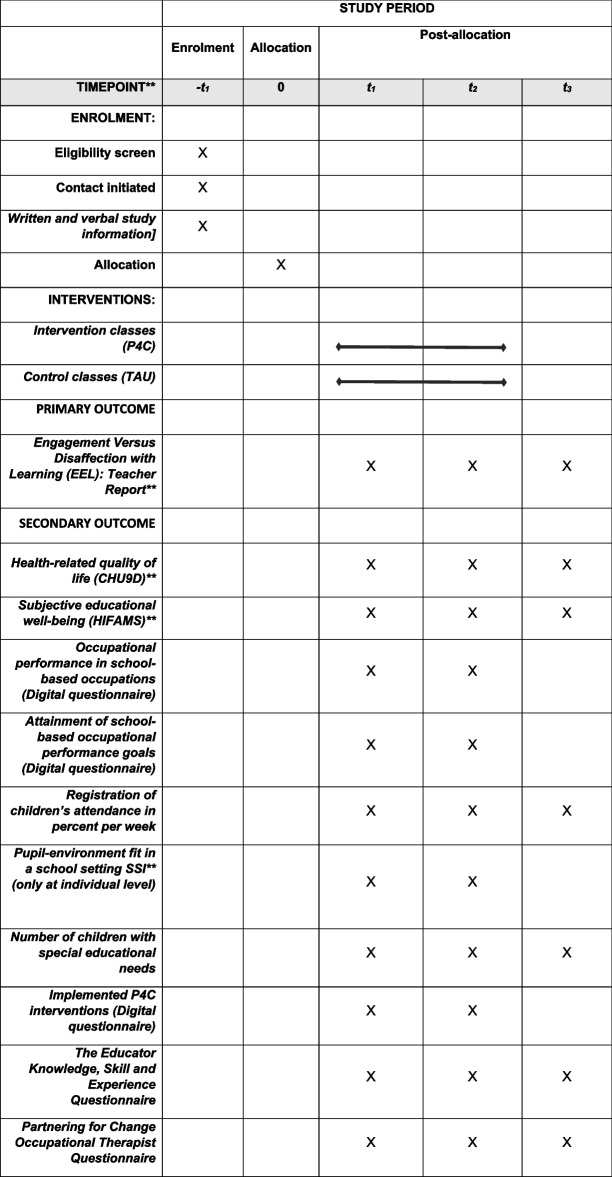
Overview of the study period, interventions, and data collection points

* T0 = Before training course in P4C; T1 = Before intervention, T2 = After intervention; T3 = Follow-up

** Engagement Versus Disaffection With Learning: Teacher Report (EEL); Swedish Child Health Utility 9D (CHU9D); How I Feel About My School (HIFAMS); School setting interview (SIS)

#### Effectiveness and cost-effectiveness of P4C

The primary outcome is children’s *engagement with learning*, measured by ‘Engagement Versus Disaffection with Learning: Teacher Report’ (EEL), focusing on children’s engagement and disaffection with learning in school [33]. The EEL consists of four subscales across 25 items, rated on a four-point Likert scale, ranging from 1 (happens almost never) to 4 (happens very often), and is rated by teachers. The subscale behavioural engagement (5 items) focuses on effort, attention, and persistence in initiating and participating in learning activities. The sub-scale of behavioural disaffection (5 items) addresses a lack of effort and withdrawal from learning. The third sub-scale—emotional engagement (5 items) —focuses on emotions indicating motivated involvement during learning activities; and emotional disaffection (9 items) addresses emotions indicating withdrawal during learning. The EEL has been shown to have good convergent validity and good test–retest reliability, and to differentiate between behavioural and emotional school engagement [4, 34]. Timeframe: baseline, four months, eleven months (both intervention and control classes).

Secondary outcomes include data on *children’s health-related quality of life (HRQOL) and wellbeing*, *children’s occupational performance*, *attainment of school-based occupational performance goals*, *school attendance, and student-environment fit*. Also, *teacher and OT knowledge and skills*, as well as *documentation of services provided* including *data on special educational needs* will be collected. Additionally, focus group interviews will be performed with school staff and children after P4C.

Children’s *HRQOL* will be measured using Swedish Child Health Utility 9D (CHU9D), a generic preference-based HRQOL measure constructed for use in children and youth. CHU9D has nine questions representing nine dimensions of HRQOL: worried, sad, pain, tired, annoyed, schoolwork/homework, sleep, daily routine, and activities [35]. Each question has five response levels representing increasing degrees of severity within each dimension and is self-completed by the child. The CHU9D has shown to be practical to use, and has acceptable face and convergent validity and internal consistency [36–39]. The CHU9D has been validated in a Swedish context and has been shown to be a feasible, reliable, and valid measures of preference based HRQOL [40]. Timeframe: baseline, four months, eleven months (both intervention and control classes).

Children’s *well-being* in school-related situations will be measured using How I Feel About My School (HIFAMS) [41]. HIFAMS addresses well-being in seven school-related situations: on the way to school, in the classroom, when doing work, at the playground, thinking about other children, thinking about their teacher, and about school in general. The HIFAMS uses a three-point Likert scale, where emotions are used to convey the responses sad (0), OK (1), and happy (2), and is self-completed by the child. The total score is calculated as the sum of the individual items (maximum total score:14), with higher scores indicating greater happiness. HIFAMS is suitable for children aged 4 to 12 years. It has shown moderate internal consistency and test–retest reliability [41, 42]. In a Swedish sample, the HIFAMS showed moderate and satisfactory internal consistency [42]. Timeframe: baseline, four months, eleven months (for both intervention and control classes).

*Occupational performance* in school-based activities will be observed by OTs and documented in a digital questionnaire created especially for the study and distributed using Survey&Report. The digital questionnaire is based on the Dynamic Performance Analysis (DPA) [31] and uses a rating scale (1–10), being 1 = no performance 10 = excellent performance. *Attainment of occupational performance goals* will be rated as attained, partly attained, or not attained before and after implemented interventions for each identified performance breakdown using the digital questionnaire. Time frame: continuously during the four-month intervention period (intervention classes).

*School attendance* per week (%) will be retrieved from the schools’ registration on children’s attendance. Time frame: baseline, four months, eleven months (for both intervention and control classes).

*Student-environment fit* in school setting will be examined only before and after individual-level interventions using the School Setting Interview (SSI) [43]. The SSI includes 16 items concerning everyday school activities, and the children’s perceived need for adjustments before and after implemented interventions. Each item is scored using a four-step rating scale; 4 = perfect fit, 3 = good fit, 2 ≤ partial fit, and 1 = unfit. The SSI has shown usability and construct validity [44]. Time frame: applied continuously during the four-month intervention period (intervention classes).

Data on the number of children who have *access to adaptions and/or special support* will be registered by teachers in each class, as will the number of children in need of additional adaptions and support. Time Frame: baseline, four months, eleven months (for both intervention and control classes).

All delivered interventions will be documented by OTs and teachers in a digital questionnaire created and distributed using Survey&Report. The type and level of interventions (class, group, individual), as well as time needed to deliver P4C each week will be documented. The questionnaire is developed for the project and has been previously used by OTs and teachers in Swedish schools. Time frame: weekly during the four-month intervention period (intervention classes).

#### School staff members’ knowledge and skills

Teachers’ knowledge, skills, and experience with P4C will be measured using the Educator Knowledge, Skill and Experience Questionnaire [27]. The questionnaire uses a five-point Likert scale from strongly disagree to strongly agree. The questionnaire has been translated into Swedish following a forward–backward translation process and previously used in Swedish elementary schools by teachers. Time frame: baseline, four months, and eleven months (both intervention and control classes).

OTs knowledge, skills, and experience of P4C will be measured using the Partnering for Change Occupational Therapist Questionnaire: Exploring Occupational Therapist Skills, Knowledge and Beliefs [27]. The questionnaire uses a five-point Likertscale from strongly disagree to strongly agree. The questionnaire has been translated into Swedish following a forward–backward translation process and has been complemented with questions relevant for the Swedish school system including questions on children with neurodevelopmental disorders. The questionnaire has been previously used in Swedish elementary schools by OTs. Time frame: baseline, four months, eleven months (for both intervention and control classes).

#### School staff members’ experience of working with P4C

Additionally, after the intervention study, focus group interviews will be conducted with school staff (OT, teacher, special education teacher and/or principal) (one focus group/school) to explore the experience of working according to P4C, and if any facilitators and barriers for implementation were identified. The interviews will be digitally recorded and transcribed verbatim.

#### Children’s’ experience of their learning environment

To describe children’s experiences of inclusive learning environments, focus group interviews will be conducted after P4C with six to eight children from intervention classes. The interviews will be digitally recorded and transcribed verbatim.

### Sample size

According to the effect size calculation for a two-sample t-test with alpha 0.05 and a power of 80% using the primary outcome (EEL), a total of 400 children should be recruited for the intervention study, 200 each in intervention and control classes, respectively (Stata v.17, StataCorp LLC, College Station, TX, USA). The calculation was based on a previous Swedish study using the EEL for sixth graders (email from Ritosa, A, PhD, andrea.ritosa@ju.se, April 2023) reporting a mean score of 3.21 and an SD of 0.74. With the above-mentioned parameters, a difference of 0.21 units in the mean between the groups can be detected, using a p-value of < 0.05 with 80% power.

### Data management

A data management plan (DMP) will be developed by the principal investigator in accordance with the Swedish National Data Service (SND). All data will be monitored regularly, entirely independently from sponsors and competing interests. Each child will be coded by school staff, and the code key connecting names to numbers will be kept in a separate, secure location at each school. All data will be securely stored on a platform for research data at Uppsala University. Access will be password protected, and only the principal investigator and co-researchers will have access to the final dataset.

### Statistical analysis

#### Effectiveness of P4C

Descriptive statistics will be presented for demographic characteristics and outcome variables. To evaluate any potential changes following the P4C, the difference-in-differences method using regression modelling will be applied [45]. This method compares the changes in outcomes over time between the intervention and control group and allows for adjustment for confounders. Furthermore, the multilevel structure of the design of the study will be considered. If the proportion of missing data is between 5% and 40%, multiple imputation will be performed [46]. Estimates will be reported with 95% confidence intervals and associated p-values. A two-tailed p-value of < 0.05 will be considered statistically significant.

#### Health economic evaluation

Cost-effectiveness analyses will be conducted as a within-trial economic evaluation comparing the health outcomes, school attendance, and societal costs between the intervention and control classes. We plan to conduct a cost-utility analysis (CUA) and cost-effectiveness analysis (CEA), which focus on the health outcomes produced, while the total society costs are related to health gains and the result is expressed as an incremental cost effectiveness ratio (ICER). The primary health outcome in economic evaluation is defined as quality adjusted life years (QALYs) gained and will be estimated using any change in utilities, measured by CHU9D instrument at baseline and respective data collection points. The change in QALYs will be estimated by multiplying the change in estimated utilities with the intervention period expressed in years. The reduction in absenteeism rate will be used as a secondary health outcome and collected during the follow-up period as well as the consumption of additional support and healthcare at school. Costs to deliver P4C will be estimated based on professionals’ time needed to deliver the intervention at school, costs of materials, and other related costs. Other societal costs such as additional support materials/equipment at school will be estimated on the group level. The total societal costs will be estimated for intervention and control classes. The cost–utility ratio will estimate the price for one additional QALY—i.e., one life year with full health gained—while the cost–effectiveness ratio will calculate the price for an avoided case of absenteeism comparing intervention and control classes. The total QALY-gain for the children during the follow-up period will be estimated using the ‘area under the curve’ method [47]. QALYs will be analysed with general regression model (GLM) [48], which allows for the control of confounders and adjustments to baseline. The costs and potential savings will be related to health gains and reduction on absenteeism, and then presented as cost–effectiveness ratios on a cost–effectiveness plane, together with uncertainty for cost- and effect data. Probability for P4C to be cost-effective compared with TAU will be presented as a range of ‘willingness-to-pay’, representing the intentions of decision-makers to pay for an additional QALY or for reduction of certain percent in absenteeism, using a ‘cost–effectiveness acceptability curve’ (CEA) [49], and bootstrap regression analysis.

#### Qualitative data analysis

The focus groups will be digitally recorded, transcribed verbatim, and analysed using content analysis [50]. The transcripts will be read through several times to get a sense of the data. To capture key thoughts or concepts, codes will be derived from the transcripts, comprising the initial coding scheme. The codes will then be organised into categories and themes highlighting the respondent’s experience.

### Ethics and dissemination

The study was granted Ethical approval by the Swedish Ethical Review Authority (2023-00013-02, 2021-06412-02, 2019-03954). Any important changes such as changes in eligibility criteria or outcome will be communicated to the relevant parties; that is participating professionals, trial registry, the Swedish Ethics Review Authority, and explicitly described in future publications. All OTs working with P4C are fully trained in the service delivery model and the study procedures. Principals, teachers, and OTs invited to participated in the study will be provided with information about P4C and the study procedure, and given time to ask questions and consider whether they wish to participate before providing verbal consent.

Informed written consent will be collected from guardians before providing individual-level interventions and for focus group interviews with children. The template provided by the Swedish Ethical Review Authority will be used for informed consent. This includes written information about the study and information stating that participants can withdraw their participation at any time. Confidentiality will be protected by using coded data on individual children with the code key being stored at each school. The data will be stored at a platform for research data at Uppsala University restricted to the research team.

Study results will be communicated through publication in scientific journals, student thesis, articles for practitioner magazines and the project website. The findings will also be presented at national and international conferences, in school settings, and within research networks.

## Discussion

Despite many efforts to support engagement with learning and participation of children at school, there is a lack of research on cost-effective, evidence-informed school-based interventions that supports children’s engagement, health, and well-being both nationally and internationally [18, 19, 51]. Enhancing children’s engagement with learning in school has been highlighted as a feasible target of intervention associated with positive outcomes across academic, social, and emotional domains [2]. Supporting children’s engagement and participation in school is stated to require service delivery models that are embedded in the natural environment that builds on interprofessional collaboration among team members [52, 53]. The hypothesis is that using P4C, a service delivery model that is embedded in the natural school setting, is based on interprofessional collaboration and incorporates a combination of class-, group- and individual-level interventions, will create an inclusive learning environment promoting engagement and well-being for all children. Given the reported need for the design of effective school-based interventions [11], this trial will inform the design and implementation of multitiered interventions targeting children’s engagement and participation in school.

### Supplementary Information

**Additional file 1: S1.** Overview of the study based on the SPIRIT Outcomes 2022 checklist

## Data Availability

Data sharing is not applicable as no datasets have yet been generated and/or analysed for this study. This is a protocol describing an intervention study design. All authors will have access to the final dataset.

## Abbreviations

P4C: Partnering for change
ADHD: Attention-deficit hyperactivity disorder
UDL: Universal design for learning
OT: Occupational therapists
PHT: Pupil Health Teams
TAU: Treatment as usual
SPIRIT: The Standard Protocol Items: Recommendations for Interventional Trials
DPA: The Dynamic Performance Analysis
EEL: Engagement Versus Disaffection with Learning: Teacher Report
HRQOL: Health-related quality of life
CHU9D: Swedish Child Health Utility 9D
HIFAMS: How I Feel About My School
SSI: The School Setting Interview
DMP: Data management plan
SND: The Swedish National Data Service
CUA: Cost-utility analysis
CEA: Cost-effectiveness analysis
ICER: Incremental cost effectiveness ratio
QALYs: Quality adjusted life years
CEA: Cost–effectiveness acceptability curve
